# Comparison of Preoperative Evaluation with the Pathological Report in Intraductal Papillary Mucinous Neoplasms: A Single-Center Experience

**DOI:** 10.3390/jcm10040678

**Published:** 2021-02-10

**Authors:** Vladimir Djordjevic, Nikica Grubor, Jelena Djokic Kovac, Marjan Micev, Natasa Milic, Djordje Knezevic, Pavle Gregoric, Zeljko Lausevic, Mirko Kerkez, Srbislav Knezevic, Dejan Radenkovic

**Affiliations:** 1 Clinic for Digestive Surgery, First Surgical Clinic, Clinical Centre of Serbia, 11000 Belgrade, Serbia; nikica.grubor@med.bg.ac.rs (N.G.); djordje.knezevic@kcs.ac.rs (D.K.); mirko.kerkez@kcs.ac.rs (M.K.); srbislav.knezevic@med.bg.ac.rs (S.K.); dejan.radenkovic@med.bg.ac.rs (D.R.); 2 Department for Surgery, Faculty of Medicine, University of Belgrade, 11000 Belgrade, Serbia; pavle.gregoric@med.bg.ac.rs (P.G.); zeljko.lausevic@med.bg.ac.rs (Z.L.); 3 Centre for Radiology and Magnetic Resonance Imaging, Clinical Centre of Serbia, 11000 Belgrade, Serbia; jelena.kovac@med.bg.ac.rs; 4 Department for Radiology, Faculty of Medicine, University of Belgrade, 11000 Belgrade, Serbia; 5 Department of Histopathology, Clinical Centre of Serbia, 11000 Belgrade, Serbia; marjan.micev@kcs.ac.rs; 6 Institute for Medical Statistics and Informatics, Faculty of Medicine, University of Belgrade, 11000 Belgrade, Serbia; milic.natasa@mayo.edu or; 7 Department of Internal Medicine, Division of Nephrology and Hypertension, Mayo Clinic, Rochester, NY 55905, USA; 8 Centre for Emergency Surgery, Clinical Centre of Serbia, 11000 Belgrade, Serbia

**Keywords:** pancreatic cystic neoplasm, intraductal papillary mucinous neoplasms, guidelines, validation

## Abstract

The key to the successful management of pancreatic cystic neoplasm (PCN), among which intraductal papillary mucinous neoplasm (IPMN) is the one with the highest risk of advanced neoplasia in resected patients, is a careful combination of clinical, radiological, and histopathological findings. This study aims to perform the comparison of a preoperative evaluation with pathological reports in IPMN and further, to evaluate and compare the diagnostic performance of European evidence-based guidelines on pancreatic cystic neoplasms (EEBGPCN) and Fukuoka Consensus guidelines (FCG). We analyzed 106 consecutive patients diagnosed with different types of PCN, among whom 68 had IPMN diagnosis, at the Clinical Center of Serbia. All the patients diagnosed with IPMNs were stratified concerning the presence of the absolute and relative indications according to EEBGPCN and high-risk stigmata and worrisome features according to FCG. Final histopathology revealed that IPMNs patients were further divided into malignant (50 patients) and benign (18 patients) groups, according to the pathological findings. The preoperative prediction of malignancy according to EEBGPCN criteria was higher than 70% with high sensitivity of at least one absolute or relative indication for resection. The diagnostic performance of FCG was shown as comparable to EEBGPCN. Nevertheless, the value of false-positive rate for surgical resection showed that in some cases, overtreating patients or treating them too early cannot be prevented. A multidisciplinary approach is essential to adequately select patients for the resection considering at the same time both the risks of surgery and malignancy.

## 1. Introduction

First described in 1824, pancreatic cystic lesions are nowadays common incidental findings on routine cross-sectional imaging owing to improvements in imaging techniques, the widespread use of various imaging modalities, and the established trend of preventive health check-ups [1]. As a heterogeneous group of pancreatic cysts, pancreatic cystic neoplasms (PCNs) include intraductal papillary mucinous neoplasms (IPMNs), mucinous cystic neoplasms (MCNs), serous cystic neoplasms (SCNs), and other rare cystic lesions such as solid pseudopapillary neoplasms (SPNs), and cystic neuroendocrine tumors (cNETs), diverging in clinical, radiological, and pathological features. In the newest WHO classification, precursor lesions including IPMN and MCN are now classified into two tiers of dysplasia rather than the three-tier system previously used, based on the highest grade of dysplasia detected [2].

A well-balanced and scientifically proven practical approach to both the diagnosis and treatment of PCN is somewhat essential, bearing in mind the necessity of balancing the cancer prevention with the risk of overtreatment, especially the surgical one. Special emphasis should be placed on IPMNs since this is the premalignant type of PCN with the highest frequency of the risk of advanced neoplasia in resected patients among PCNs and hence, requires either surveillance or in some cases, surgical resection [3,4,5]. It is characterized by papillary growths within the pancreatic ductal system with thick mucin secretion with the present risk of undergoing malignant transformation [6]. The true incidence of IPMNs is unknown since most of them are small and asymptomatic. However, it has been estimated that the frequency is approximately 2.6–13.5% and the age of presentation is typically between the 5th and 7th decade [7]. The question of whether surgical resection is necessary for such patients remains one of the biggest surgical challenges nowadays. There are reports of IPMNs progression with no concerning morphology up to 16 years of follow-up [8]. The current understanding points to a good prognosis based on the early identification and proper management in a multidisciplinary. Hence, several guidelines have been developed to ensure the best approach in IPMN and PCN management in general. Interestingly, however, apart from many similarities between them, still there are some differences and controversial topics. As recently reviewed by van Huijgevoort et al. [5], there are currently three guidelines providing recommendations on PCN management based on symptoms and perceived risk of malignancy. In 2015, the American Gastroenterological Association (AGA) provided guidelines suggesting that a solid component and a dilated pancreatic duct and/or cytology positive for malignancy is an indication for the surgery. These guidelines also recommended the discontinuation of the patients’ surveillance in the case of no significant change in the cyst during 5 years of follow-up [9]. In 2012, the International Association of Pancreatology (IAP) updated its 2006 guidelines [10] providing a fresh insight into the risk stratification, based on extensive new research data, articulating high-risk stigmata and worrisome features of Fukouka Consensus Guidelines (FCG) [11]. Namely, immediate resection is recommended in the case of high-risk features, while in the case of worrisome features the presence of a conservative approach is recommended. The FCG were slightly revised and updated in 2017 along with new literature findings [3]. Three factors were described as high-risk stigmata, obstructive jaundice, enhancing of a mural nodule ≥ 5 mm, and a main pancreatic duct (MDP) ≥ 10 mm. Any of these factors is an absolute indication for surgery. Additionally, the presence of any of the nine worrisome features is considered to be an indication for endoscopic ultrasonography (EUS), (cyst size ≥ 3 cm, acute pancreatitis, thicken, enhancing walls, dilated duct (5 to 9 mm), non-enhancing mural nodule, change in duct caliber with distal atrophy, lymphadenopathy). The European response to the Tanaka et al. [11] was given by the European Consensus. The European Study Group on Cystic Tumors of the Pancreas in 2013 provided Expert Consensus with a clear distinction between absolute and relative indications for surgery and defined the surveillance intervals (6 months during the first year, yearly afterwards) [12]. The European Consensus were revised in 2018 with the first evidence-based guidelines on the management of PCN, European evidence-based guidelines on pancreatic cystic neoplasms (EEBGPCN) [4]. These guidelines introduced new relative indicators for the resection: Growth rate > 5 mm per year, new-onset diabetes mellitus, and acute pancreatitis caused by IPMN. The importance of appropriate surgical strategies in the IPMN treatment has been highlighted [13], together with the plea on including the surgery type in the decision-making algorithm [14]. Results from the study by McGinnis et al. [15] on 72 pancreatic adenocarcinoma tumor resection patients showed that patients with pancreatic adenocarcinoma arising from IPMNs have a statistically significant longer overall survival and progression-free survival when compared to patients with tumors arising from pancreatic intraepithelial neoplasms, which highlight the importance of the appropriate surgical management of IPMN. A number of studies have been dealing with the variety of preoperative factors for determining the prognosis in pancreatic cancer [16,17], having in mind that pancreatic cancer has been lagging in the overall success of identifying these factors in other types of cancers. Similarly, knowledge of the risk factors that can be connected to pancreatic cancer has been mounting recently [18,19,20] together with one on the molecular basis of cancer, paving the way for new and more effective potential therapeutic targets for cancer in general [21,22].

The key to the successful management of IPMN is a careful combination of clinical, radiological, and histopathological findings in order to establish the appropriate diagnostic and follow-up strategies and clearly defined indications for surgery. This study aims to perform the comparison of preoperative evaluation with a pathological report in IPMN based on a single-center experience to evaluate and compare the diagnostic performance of EEBGPCN and Fukuoka guidelines.

## 2. Materials and Methods

### 2.1. Study Population

We analyzed and reviewed the medical records of 68 IPMN patients among 106 consecutive patients diagnosed with different PCNs (IPMNs, MCNs, SCNs, SPNs, and cNETs) at the Clinical Center of Serbia, Belgrade, Serbia between January 2012 and December 2020. Patients were treated using the standard surgical treatment for resectable pancreatic cancer: Whipple procedure (pancreaticoduodenectomy), distal pancreatectomy, or total pancreatectomy. Detailed clinical information on the enrolled participants was collected including age at the time of surgery, gender, presence of symptoms (jaundice and history of acute pancreatitis), presence of DM, new-onset DM, and preoperative CA 19-9 serum levels, imaging studies, surgical and pathologic reports. Preoperative imaging diagnosis was made by endoscopic ultrasonography (EUS), multi-sliced computed tomography (MSCT), magnetic resonance imaging (MRI), and/or endoscopic retrograde cholangiopancreatography (ERCP) after aspiration of pure pancreatic juice. The cyst, mural nodule sizes, and diameter of the main pancreatic duct were determined by imaging studies recording the maximum measurements. The surgical pathology specimens were reviewed by an experienced pathologist. All the patients with IPMNs were further stratified concerning the presence of the absolute (positive cytology for malignancy/high-grade dysplasia, solid mass, jaundice) and relative indications (enhancing mural nodule > 5 mm, MPD dilation ≥ 10 mm, grow rate ≥ 5 mm/year, increased levels of serum CA 19-9 > 37 U/mL, MPD dilation between 5 and 9.9 mm, cyst diameter ≥ 40 mm, new-onset DM, acute pancreatitis, enhancing mural nodule < 5 mm for resection) according to EEBGPCN. The classification of IPMN patients also included FCG, namely, high-risk stigmata and worrisome features. Patients with branch duct (BD)-IPMN were analyzed separately, as well. According to the pathological findings patients were divided into benign, low-grade dysplasia (LGD), and malignant, high-grade dysplasia/invasive carcinoma (HGD/IC) groups. Demographic data, radiological findings, and clinical characteristics between these two groups were compared in order to reveal the accuracy of EEBGPCN and FCG in the prediction of malignancy in our series of IPMN patients. The study protocol was approved by the Ethical boards of the Clinical Center of Serbia and School of Medicine, the University of Belgrade (no. 1322/11-8).

### 2.2. Statistical Analysis

Numerical data are presented as means with standard deviations, while categorical variables are summarized by absolute numbers with percentages. Differences in demographic data and clinical characteristics between LGD and HGD/IC groups were assessed by the Student’s t-test for numerical data and the chi-square test for categorical variables. Fisher’s exact test was used when the cell counts were fewer than 5. Sensitivity, specificity, positive predictive value (PPV), and negative predictive value (NPV) were calculated to evaluate the performance of European evidence-based guidelines and Fukuoka consensus guidelines on pancreatic cystic neoplasms in identifying HGD/IC. The multivariate logistic regression analysis was used to identify the most important predictors of high-grade dysplasia/invasive carcinoma. Significant variables from the univariate logistic regression analysis were entered into the multivariate analysis in all models. Results are presented as odds ratios with corresponding 95% confidence intervals. In all the analyses, the significance level was set at 0.05. Statistical analysis was performed using the IBM SPSS statistical software (SPSS for Windows, release 25.0, SPSS, Chicago, IL, USA).

## 3. Results

### 3.1. Patient Characteristics

Among the 106 patients included in the study, 68 had IPMN diagnosis, MCNs 14, SCNs 17, SPNs five, and cNETs two. The distribution of patients by diagnosis and final confirmation of malignancy presence based on surgical pathology is shown in Table 1.

The clinical characteristics of the IPMN study population stratified by final surgical pathology results are shown in Table 2. Among these 68 patients, 50 (73.5%) patients were classified as high-grade dysplasia (HGD)/invasive carcinoma (IC), which included 14 HGD and 36 IC, the remaining 18 (26.5%) had a low-grade dysplasia (LGD) diagnosed by surgical pathology. The mean age of these 68 patients was 60.79 years (34–78 years) with a male: Female ratio of 1.12:1. Most of the lesions were located at the pancreatic head (60.3%, 41/68) and were mixed type IPMNs (47.1%, 32/68). The main duct IPMN (MD-IPMN) and mixed type IPMN have a significantly higher percentage of HDG/IC cases when compared to BD-IPMN (*p* = 0.005) (Table 2). Indications for resection according to the EEBGPCN stratified by final surgical pathology results are shown in Table 3. Among these 68 patients, the IPMN type, as well as solid mass and jaundice (as absolute indications for resection), and increased levels of serum CA 19-9 > 37 U/mL (as a relative indication) were significantly different between the LGD and HGD/IC groups (Table 3). In the multivariate logistic regression analysis, CA 19-9 > 37 U/mL was identified as an independent parameter of invasive IPMN (*p* < 0.001; OR = 21.5; 95% CI 4.3–106.9). Among the 25 patients with BD-IPMN, 12 (48.0%) had an LGD diagnosis by surgical pathology. The remaining 13 (52.0%) patients were classified as HGD/IC, which included five HGD and eight IC based on surgical pathology (Table 2). The mean age of these 25 patients was 57.72 years (35–77 years) with a male:female ratio of 1:1.27. Most of the lesions were located at the pancreatic head (44.0%, 11/25) (Table 2). Among these 25 patients, the gender and increased levels of serum CA 19-9 > 37 U/mL (relative indication) were significantly different between the LGD and HGD/IC groups (Table 2 and Table 3).

Indications for resection according to the Fukuoka consensus guidelines on pancreatic cystic neoplasms stratified by final surgical pathology results are shown in Table 4. In all IPMNs, among high-risk stigmata indications for resection, obstructive jaundice was significantly different between the LGD and HGD/IC groups. Among worrisome feature indications for resection, an elevated Ca 19-9 was significantly different between the LGD and HGD/IC groups. In the multivariate logistic regression analysis, both obstructive jaundice and CA 19-9 > 37 U/mL were identified as significant predictors of invasive IPMN, and CA 19-9 > 37 U/mL was found to be an independent parameter (*p* < 0.001; OR = 55.5; 95% CI 6.3–490.1). In the BD-IPMN subgroup, among high-risk stigmata indications for resection, there were no significantly different indications between the LGD and HGD/IC groups. Among worrisome feature indications for resection, an elevated Ca 19-9 was significantly different between the LGD and HGD/IC groups. When European evidence-based guidelines were applied to the study population, 52 (76.5%, 52/68) patients met the criteria with at least one absolute indication for resection, and out of them 41 (78.8%, 41/52) were verified with HGD/IC (Table 3). In the patients with at least one relative indication for resection, 47 of 64 (73.4%) patients were verified with HGD/IC. Among the patients with BD-IPMN, 17 (68.0%) patients met the criteria with at least one absolute indication for resection, and 11 (64.7%) were verified with HGD/IC (Table 3). In the patients with at least one relative indication for resection, 12 of 24 (50.0%) patients were verified with HGD/IC.

When Fukuoka consensus guidelines were applied to the study population, 42 (61.8%, 42/68) patients met the criteria with at least one absolute high-risk stigmata indication for resection, and out of whom 34 (81.0%) were verified with HGD/IC (Table 4). All the patients had at least one worrisome feature. Among the patients with BD-IPMN, 11 (44.0%) patients met the criteria with at least one high-risk stigmata indication for resection, and eight (72.7%) were verified with HGD/IC (Table 4).

### 3.2. Diagnostic Performance of the European Evidence-Based and Fukuoka Consensus Guidelines for Resected IPMNs

Based on the final surgical pathology, Table 5 shows the diagnostic performance of indications for resection according to the European evidence-based guidelines stratified by all patients with IPMN and patients with BD-IPMN. The sensitivity, PPV, specificity, NPV, and accuracy for at least one absolute indication for resection according to the European evidence-based guidelines to identify HGD/IC in all the patients with IPMN were 82.0%, 78.8%, 38.9%, and 43.8%, respectively. The diagnostic performance for at least one absolute and at least one relative indication for resection among all IPMNs were 80.0%, 80.0%, 44.4%, and 44.4%, respectively. Table 5 also shows the diagnostic performance of at least one relative indication and at least one absolute or one relative indication for resection according to the European evidence-based guidelines. In addition, the diagnostic performance of increased levels of serum CA 19-9 is presented (74.5%, 94.6%, 89.5%, and 58.6%).

Table 6 shows the diagnostic performance of indications for resection according to the Fukuoka consensus guidelines stratified by all the patients with IPMN and patients with BD-IPMN. The sensitivity, PPV, specificity, NPV, and accuracy for at least one high-risk stigmata presence according to the Fukuoka consensus guidelines to identify HGD/IC in all the patients with IPMN were 68.0%, 81.0%, 55.6%, and 38.5%, respectively. Table 5 also shows the diagnostic performance of at least one absolute and one relative indication for resection according to the Fukuoka consensus guidelines (68.0%, 81.0%, 55.6%, and 38.5%). The diagnostic performance of at least one worrisome feature presence is not presented as all the patients have at least one worrisome feature.

Both the diagnostic performance of indications for resection according to the European evidence-based guidelines and Fukuoka consensus guidelines are also presented for BD-IPMN (Table 5 and Table 6).

## 4. Discussion

The IPMN type of PCN is premalignant and hence, requires either surveillance or in some cases, surgical resection [3,4]. However, the discussion on the necessity of surgical resection remains one of the biggest surgical challenges nowadays. This is the case mainly since the surgical resection of IPMN is associated with surgical morbidity/mortality rates, while not all IPMN are malignant. Further, it should be noted that the risk of advanced neoplasia in resected IPMN patients has a mean frequency of 62% which is the highest among other PCNs [5]. In comparison to the 2017 Fukuoka IAP guidelines [3] and 2018 European guidelines [4], 2015 AGA guidelines are somewhat more conventional recommending the resection for IPMN cases, but only in the presence of a nodule or cytology positive for malignancy [9]. As stated in the recent review by van Huijgevoort et al. [5], although more conservative, and hence allowing fewer patients to undergo an unnecessary operation, the risk of missing patients with advanced neoplasm following AGA guidelines is high. Hence, in the present study, we have decided to study and compare the diagnostic performance of the 2017 IAP Fukuoka guidelines and 2018 EEBGPCN guidelines for IPMN management.

In a case series of 68 pathologically proven IPMNs, we have determined the frequency of HGD/IC cases to be 73.5%. These values were somewhat higher than those observed in the available literature data. Namely, the study which included 230 consecutive patients who underwent surgery for IPMN revealed 33.5% of patients with HGD/IC pathology [23]. Similarly, in a study with 158 patients enrolled, a 32.3% frequency of HGD/IC was observed [24]. However, the obtained percentage is within the interval given in the review paper by van Huijgevoort et al. [5]. In the present study, the age of presentation and observed gender distribution follows key demographic features of IPMN patients [5].

Moreover, our study has shown that among the patients diagnosed with BD-IPMN, a significantly higher percentage has been diagnosed with LGD, while in MD-IPMN and mixed type IPMN patients, a significantly higher percentage of HGD/IC cases was presented. These observations are in accordance with the literature data [25,26,27,28]. Among the 25 patients diagnosed with BD-IPMN, 11 (84.6%) and 12 (92.3%) out of 13 patients with pathologically verified HGC/IC have met EEBGPCN guidelines of at least one absolute and at least one relative indication, respectively. However, none of the 13 patients presumed not to meet any of the criteria for the resection to be later verified with HGD/IC giving the missing rate for EEBGPCN to be as low as 0%. This was under the high sensitivity of at least one absolute or relative indication for resection according to EEBGPCN. The high sensitivity of the same parameter was also noticed in the study by Jan et al. [24] in which 158 consecutive patients with IPMN diagnosis at the National Taiwan University Hospital were retrospectively analyzed and reviewed. The same study revealed a false-positive rate for surgical resection according to the same parameter, with 58.3% in patients with resected BD-IPMN, while the false-negative rate was 10%. In the present study, these rates were 50% and 0%, respectively. It can be postulated that even though EEBGPCN presents less rigorous criteria than other guidelines intended for IPMN management, overtreating patients or treating them too early during the disease course, nevertheless, cannot be avoided. On the other hand, a recent prospective cohort study performed on 128 patients with IPMN highlighted the importance of surveillance time not only in the sense of cyst progression, but also in terms of changes in the patients’ conditions making them during time unfit for surgery. However, the study has shown the surveillance of BD-IPMN according to EEBGPCN as feasible [29].

Among the absolute indicators, the presence of jaundice and solid mass were shown to be significantly associated with malignancy and were proven to be the predictors of IPMN with HGD/IC, which agrees with the available literature data [14,24,30]. Although relatively new criteria was applied for the IPMN management and listed as a relative indication for the resection according to EEBGPCN, the present study confirmed increased levels of serum CA 19-9 as predictors of HGD/IC. Generally perceived as a prognostic factor in patients with pancreatic cancer [31], its value in the IPMN prognosis has been recently revealed. In a study by Jan et al. [24], increased levels of this marker were also shown to accurately predict HGD/IC among the resected IPMN group. The value of this parameter as a predictive factor for IPMN with HGD/IC was also highlighted in a systematic review performed by Heckler et al. [30]. Our study recommends CA 19-9 as a valuable supplement in IPMN management and highlights its use as a relative indication for IPMN resection, as recommended by the EEBGPCN. Particularly, the use of laboratory data such as serum CA 19-9 levels as an indication for resection is one of the main differences between EEBGPCN and other guidelines in use.

Similarly, to the absolute indications for surgery given by EEBGPCN, the revised Fukuoka IAP guidelines described three factors which are high-risk stigmata as being the absolute surgical indications [3]. On the other hand, worrisome features are regarded as an indication for EUS. The calculated PPV for at least one high-risk stigmata was 81% for all IPMNs cases and 72.7% for BD-IPMN, keeping the false-positive rate under 30%. Contrastingly, a false-negative rate was higher, especially when all the IPMNs cases were studied. High-risk stigmata were also shown to be capable of identifying HGD/IC IPMNs patients, especially in the BD-IPMN group. Among these parameters, the presence of obstructive jaundice was associated with the statistically higher number of cases with HGD/IC diagnosis among all the IPMNs cases. The supremacy of this very parameter was shown in a study conducted on 230 patients with IPMN [23]. The performance of worrisome features as a parameter in identifying HGD/IC could not be assessed in our study since all the patients have at least one worrisome feature. However, an elevated CA 19-9 was once again identified as an independent parameter of invasive IPMN in all the IPMN patients and those with the BD type, as well. In a retrospective analysis of 1369 patients with BD-IPMN from Seoul National University Hospital in Korea for the period 2001–2016, most cysts were found to be indolent, but some rapidly grew and progressed pointing to the need for individualized surveillance protocols for these patients [32].

Certain limitations of the study must be outlined. First, the study involved a small number of patients. Further, all the patients received surgical treatment, thus the outcomes of the study might not be applied to the patients evaluated before surgical resection. Due to a small number of patients involved, some of the statistical parameters could not be calculated. Finally, detailed follow-up histories were not obtained. Furthermore, large population-based prospective studies are essential to completely evaluate the performance of these guidelines.

## 5. Conclusions

Although a relatively small sample size was studied, based on the obtained results in the study, we can conclude that the diagnostic performance of the European evidence-based guidelines and Fukuoka consensus guidelines on pancreatic cystic neoplasms in identifying HGD/IC IPMNs is comparable. Both absolute and relative indications of the European evidence-based guidelines for the resection of IPMNs showed statistically significant differences between the LGD and HGD/IC groups, with absolute indications being superior. Among the Fukuoka consensus guidelines indications, high-risk stigmata were superior to the worrisome features indications in identifying HGD/IC IPMNs. Increased levels of serum CA 19-9 were identified as the most important relative indication for surgical resection of IPMNs for all the IPMNs types. Moreover, it could be concluded that PCN still represents a diagnostic challenge. A multidisciplinary approach is essential to adequately select patients for the resection, while at the same time considering both the risks of surgery and malignancy. Further studies should be directed towards this precedence.

## Figures and Tables

**Table 1:** Distribution of patients by type of pancreatic cystic neoplasm, basic demographics, and final surgical pathology confirmation of malignancy presence.

Type	Age	Male/Female	HGD/IC (%)
IPMN	60.79 ± 10.27 (34–78)	36/32	50 (73.5)
MCN	47.23 ± 14.01 (27–79)	1/13	1 (7.1)
SCN	60.71 ± 17.03 (30–89)	3/14	0 (0)
SPN	32.00 ± 12.73(23–41)	0/5	0 (0)
cNET	70.50 ± 0.70 (70–71)	1/1	0 (0)

Abbreviations: IPMN: intraductal papillary mucinous neoplasm; MCN: mucinous cystic neoplasms; SCN: serous cystic neoplasms; SPN: solid pseudopapillary neoplasms; cNET: cystic neuroendocrine tumors; HGD/IC: high-grade dysplasia/invasive carcinoma. The age of patients is represented as the mean ± standard deviation (SD); min-max.

**Table 2:** Demographic data of all the patients with papillary mucinous neoplasm and patients with branch duct-IPMN.

	All IPMN (*n* = 68)			BD-IPMN (*n* = 25)		
	LGD *n =* 18	HGD/IC *n =* 50	*p*-Value	LGD *n =* 12	HGD/IC *n =* 13	*p*-Value
Age, years (mean ± SD)	61.33 ± 12.12	60.60 ± 9.65	0.797	56.33 ± 11.37	59.0 ± 9.87	0.537
Gender, male (*n*, %)	11 (57.9)	25 (51.0)	0.418	8 (66.7)	3 (23.1)	0.028
Tumor location (*n*, %)						
Head	10 (55.6)	31 (62.0)	0.147	6 (50.0)	5 (38.5)	0.270
Body	5 (27.8)	7 (14.0)		4 (33.3)	2 (15.4)	
Tail	3 (16.7)	4 (8.0)		2 (16.7)	3 (23.1)	
>1 pancreatic location involved	0 (0)	8 (16.0)		0 (0)	3 (23.1)	
IPMN type (*n*, %)						
Main	3 (16.7)	8 (16.0)	0.947			
Branch	12 (66.7)	13 (26.0)	0.002			
Mixed	3 (16.7)	29 (58.0)	0.003			

Abbreviations: LGD: low-grade dysplasia; HGD/IC: high-grade dysplasia/invasive carcinoma; IPMN: Intraductal papillary mucinous neoplasm; BD-IPMN: branch duct-IPMN. Differences between LGD and HGD/IC were assessed by the Student’s t-test for numerical data and Chi-square/Fisher’s exact test for categorical variables.

**Table 3:** Indications for resection according to the evidence-based guidelines on pancreatic cystic neoplasms (EEBGPCN).

	All IPMN (*n* = 68)			BD-IPMN (*n* = 25)		
	LGD *n =* 18	HGD/IC *n =* 50	*p*-Value	LGD *n =* 12	HGD/IC *n =* 13	*p*-Value
At least one absolute indication for resection (*n*, %)	11 (61.1)	41 (82.0)	0.073	6 (50.0)	11 (84.6)	0.097
Positive cytology for malignancy/HGD (*n*, %)	0 (0.0)	1 (2.1)	1.000	0 (0.0)	0 (0.0)	NA
Solid mass (*n*, %)	6 (33.3)	36 (72.0)	0.004	4 (33.3)	8 (61.5)	0.158
Jaundice (*n*, %)	4 (22.2)	26 (54.2)	0.020	2 (16.7)	5 (41.7)	0.371
Enhancing mural nodule > 5 mm (*n*, %)	3 (16.7)	10 (20.4)	0.731	2 (16.7)	4 (30.8)	0.645
MPD dilation ≥ 10 mm (*n*, %)	4 (22.2)	14 (28.6)	0.603	1 (8.3)	3 (23.1)	0.593
At least one relative indication for resection (*n*, %)	17 (94.4)	47 (95.9)	0.796	12 (100)	12 (92.3)	1.000
Grow rate ≥ 5 mm/year (*n*, %)	0 (0.0)	1 (2.1)	1.000	0 (0.0)	0 (0.0)	NA
Increased levels of serum CA 19-9 > 37 U/mL (*n*, %)	2 (11.1)	35 (72.9)	<0.001	1 (8.3)	7 (58.3)	0.027
MPD dilation between 5 and 9.9 mm (*n*, %)	12 (66.7)	32 (66.7)	1.000	9 (75.0)	9 (75.0)	1.000
Cyst diameter ≥ 40 mm (*n*, %)	5 (27.8)	23 (46.9)	0.159	4 (33.3)	6 (46.2)	0.688
New-onset DM (*n*, %)	1 (5.6)	5 (10.4)	0.541	1 (8.3)	1 (8.3)	1.000
Acute pancreatitis	1 (5.6)	5 (10.4)	0.541	1 (8.3)	1 (8.3)	1.000
Enhancing mural nodule < 5 mm (*n*, %)	7 (38.9)	23 (46.9)	0.557	4 (33.3)	4 (30.8)	1.000

Abbreviations: CA: Carbohydrate antigen; DM: Diabetes mellitus; LGD: low-grade dysplasia; HGD/IC: high-grade dysplasia/invasive carcinoma; IPMN: Intraductal papillary mucinous neoplasm; MPD: Main pancreatic duct. Differences between LGD and HGD/IC were assessed by Chi-square/Fisher’s exact test for categorical variables.

**Table 4:** Indications for resection according to the Fukuoka consensus guidelines on pancreatic cystic neoplasms.

	All IPMN (*n* = 68)			Branch Duct-IPMN (*n* = 25)		
	LGD *n =* 18	HGD/IC *n =* 50	*p*-Value	LGD *n =* 12	HGD/IC *n =* 13	*p*-Value
At least one high-risk stigmata indication for resection	8 (44.4)	34 (68.0)	0.078	3 (25.0)	8 (61.5)	0.066
Obstructive jaundice	4 (22.2)	29 (58.0)	0.009	2 (16.7)	6 (46.2)	0.202
Enhancing solid component > 5 mm	3 (16.7)	14 (28.0)	0.527	2 (16.7)	6 (46.2)	0.202
Main pancreatic duct ≥ 10 mm	3 (16.7)	16 (32.0)	0.214	0 (0.0)	3 (23.1)	0.220
At least one worrisome feature	18 (100.0)	50 (100.0)		12 (100.0)	13 (100.0)	
Size ≥ 3cm	7 (38.9)	31 (62.0)	0.090	5 (41.7)	7 (53.8)	0.543
Enhancing mural nodule < 5 mm	7 (38.9)	23 (46.0)	0.602	4 (33.3)	4 (30.8)	1.000
Thickened and enhancing cyst wall	8 (44.4)	34 (68.0)	0.078	5 (41.7)	8 (61.5)	0.320
Main pancreatic duct 5–9 mm	13 (72.2)	33 (66.0)	0.628	10 (83.3)	10 (76.9)	1.000
Elevated Ca 19-9	2 (11.1)	35 (72.9)	<0.001	1 (8.3)	7 (58.3)	0.027
Cyst growth rate ≥ 5 mm in 2 years	0 (0.0)	2 (4.3)	1.000	0 (0.0)	0 (0.0)	NA
Abrupt change in caliber of the pancreatic duct with distal pancreatic atrophy	1 (5.6)	5 (10.0)	0.569	0 (0.0)	2 (15.4)	0.480
Lymphadenopathy	8 (44.4)	30 (60.0)	0.254	5 (41.7)	8 (61.5)	0.320

Abbreviations: LGD: low-grade dysplasia; HGD/IC: high-grade dysplasia/invasive carcinoma; IPMN: Intraductal papillary mucinous neoplasm. Differences between LGD and HGD/IC were assessed by Chi-square/Fisher’s exact test for categorical variables.

**Table 5:** Diagnostic performance of specified indications for resection according to the European evidence-based guidelines on pancreatic cystic neoplasms stratified by patients with intraductal papillary mucinous neoplasms and patients with branch duct-intraductal papillary mucinous neoplasms.

	All IPMN (*n* = 68)				BD-IPMN (*n* = 25)			
	Sensitivity	PPV	Specificity	NPV	Sensitivity	PPV	Specificity	NPV
At least one absolute indication	82.0%	78.8%	38.9%	43.8%	84.6%	64.7%	50.0%	75.0%
At least one absolute and one relative indications	80.0%	80.0%	44.4%	44.4%	84.6%	64.7%	50.0%	75.0%
At least one relative indication	95.9%	73.4%	5.6%	33.3%	92.3%	50.0%	0.0%	0.0%
At least one absolute or one relative indication	96.0%	72.7%	0.0%	0.0%	92.3%	50.0%	0.0%	0.0%
Increased levels of serum CA 19-9	72.9%	94.6%	88.9%	55.2%	58.3%	87.5%	91.7%	68.8%

Abbreviations: IPMN: Intraductal papillary mucinous neoplasm; BD-IPMN: branch duct-IPMN.

**Table 6:** Diagnostic performance of indications for resection according to the Fukuoka consensus guidelines on pancreatic cystic neoplasms stratified by patients with intraductal papillary mucinous neoplasms and patients with branch duct-intraductal papillary mucinous neoplasms.

	All IPMNs (*n* = 68)				BD-IPMN (*n* = 25)			
	Sensitivity	PPV	Specificity	NPV	Sensitivity	PPV	Specificity	NPV
At least one high-risk stigmata	68.0%	81.0%	55.6%	38.5%	61.5%	72.7%	75.0%	64.3%
At least one high-risk stigmata and one worrisome feature	68.0%	81.0%	55.6%	38.5%	61.5%	72.7%	75.0%	64.3%
At least one worrisome feature	NA	NA	NA	NA	NA	NA	NA	NA
At least one high-risk stigmata or one worrisome feature	NA	NA	NA	NA	NA	NA	NA	NA

Abbreviations: IPMN: Intraductal papillary mucinous neoplasm; BD-IPMN: branch duct-IPMN; NA: Not applicable (all the patients have at least one worrisome feature).

## Data Availability

The data presented in this study are available on request from the corresponding author.
